# Probiotics Differently Affect Gut-Associated Lymphoid Tissue Indolamine-2,3-Dioxygenase mRNA and Cerebrospinal Fluid Neopterin Levels in Antiretroviral-Treated HIV-1 Infected Patients: A Pilot Study

**DOI:** 10.3390/ijms17101639

**Published:** 2016-09-27

**Authors:** Carolina Scagnolari, Giuseppe Corano Scheri, Carla Selvaggi, Ivan Schietroma, Saeid Najafi Fard, Andrea Mastrangelo, Noemi Giustini, Sara Serafino, Claudia Pinacchio, Paolo Pavone, Gianfranco Fanello, Giancarlo Ceccarelli, Vincenzo Vullo, Gabriella d’Ettorre

**Affiliations:** 1 Istituto Pasteur Italia, Fondazione Cenci Bolognetti, Viale Regina Elena 291, 00161 Rome, Italy; carolina.scagnolari@uniroma1.it (C.S.); claudiapinacchio@gmail.com (C.P.); 2 Department of Molecular Medicine, Laboratory of Virology, Sapienza University of Rome, Viale di Porta Tiburtina 28, 00185 Rome, Italy; carla.selvaggi@uniroma1.it; 3 Department of Public Health and Infectious Diseases, Sapienza University of Rome, Viale del Policlinico 155, 00161 Rome, Italy; giuseppe.coranoscheri@uniroma1.it (G.C.S.); schietroma.ivan@gmail.com (I.S.); saeednajafifard@yahoo.com (S.N.F.); andrea.m91@hotmail.it (A.M.); noemi.giustini@uniroma1.it (N.G.); sara.serafino@uniroma1.it (S.S.); pavopaolo@gmail.com (P.P.); giancarlo.ceccarelli@uniroma1.it (G.C.); vincenzo.vullo@uniroma1.it (V.V.); 4 Department of Emergency Surgery, Emergency Endoscopic Unit, Policlinico Umberto I, Sapienza University of Rome, Viale del Policlinico 155, 00161 Rome, Italy; gianfranco.fanello@endoroma.it

**Keywords:** HIV-1, IDO, neopterin, tryptophan, HAND, CNS

## Abstract

Recently the tryptophan pathway has been considered an important determinant of HIV-1 infected patients’ quality of life, due to the toxic effects of its metabolites on the central nervous system (CNS). Since the dysbiosis described in HIV-1 patients might be responsible for the microbial translocation, the chronic immune activation, and the altered utilization of tryptophan observed in these individuals, we speculated a correlation between high levels of immune activation markers in the cerebrospinal fluid (CSF) of HIV-1 infected patients and the over-expression of indolamine-2,3-dioxygenase (IDO) at the gut mucosal surface. In order to evaluate this issue, we measured the levels of neopterin in CSF, and the expression of IDO mRNA in gut-associated lymphoid tissue (GALT), in HIV-1-infected patients on effective combined antiretroviral therapy (cART), at baseline and after six months of probiotic dietary management. We found a significant reduction of neopterin and IDO mRNA levels after the supplementation with probiotic. Since the results for the use of adjunctive therapies to reduce the levels of immune activation markers in CSF have been disappointing so far, our pilot study showing the efficacy of this specific probiotic product should be followed by a larger confirmatory trial.

## 1. Introduction

A key role of tryptophan metabolism in causing the chronic immune activation status in patients with HIV-1 infection has been proposed [1,2]. Altogether, these aspects are relevant in the context of the contribution of the microbiota composition in modulating the gut–brain axis (GBA) [3]. The discovery that differential gut microbial composition is associated with alterations in behavior and neurocognitive functions has significantly contributed to establish the “microbiota–gut–brain-axis” as an extension of the well-accepted GBA concept [4]. The latter is generally used to describe the existence of a bidirectional communication network between the CNS and intestinal organs, including the enteric nervous system (ENS) [3]. Here, we speculate that the presence of a dysfunction in tryptophan metabolism during HIV-1 infection may contribute to a disruption of the integrity of the intestinal mucosal barrier, worsen microbial translocation, and, indirectly, immune activation status. In particular, the gut associated lymphoid tissue (GALT) is considered to be one of the preferential sites of HIV-1 replication, and members of the class Clostridia have been found to induce mucosal expression of indolamine-2,3-dioxygenase (IDO) [5]. Directly related to this, HIV-1 infected patients are likely to harbor enteropathogenic bacteria that can catabolize tryptophan into immunomodulatory kynourenine derivatives, known to correlate with disease progression and mucosal immune disruption [6]. Indeed, it has been recently reported that HIV-1 infection leads to a significant and distinct impact on the metabolism of the gut ecosystem compared with other diseases [7]. In the course of HIV-1 infection, the chronic immune activation—partially due to a dysfunction of the GALT—with consequent microbial translocation has been linked with an increased production of interferon (IFN) gamma and IDO activity [8,9]. IDO is an enzyme involved in tryptophan metabolism, and its expression is increased by many cytokines. This enzyme can induce an increase in kinurenine and other metabolites, such as quinolinic acid. Those molecules have been reported to be associated with the neuronal toxicity and neurocognitive disorders in HIV-1 infected patients [9,10,11,12,13]. Moreover, kynurenine can act as negative regulators of T cell proliferation [14], while quinolinic acid is related to the onset of neurocognitive disorders, such as the AIDS dementia complex [2]. Notably, IDO has been also associated with a reduction in Th17 cell frequency, which plays a pivotal role in the maintenance of gut mucosal integrity during HIV-1 infection [15,16]. Given the strong relationship between the damage of the intestinal epithelium and the presence of altered gut microbiota composition in HIV-1 population, and the hypothetical link with the mucosal expression of IDO, the administration of oral probiotics in combination with combined antiretroviral therapy (cART) could limit the outgrowth of pathobionts that exacerbate mucosal immune disequilibrium in IDO expression. Neopterin—produced by macrophages upon IFN gamma stimulation—is a recognized marker of monocyte activation, and an association between its expression and the development of HIV-associated neurocognitive disorders (HAND) has been proposed [17,18,19,20]. The expression of neopterin has been found to be higher both in naïve HIV-1-positive patients compared to those on cART and in HIV-1 infected patients on effective cART than in healthy people [20,21]. In light of the above considerations, we performed the first longitudinal pilot study evaluating the effects of a high concentration multistrain probiotic product on neopterin levels in cerebrospinal fluid (CSF) and on the expression of IDO mRNA measured in lymphocytes of lamina propria (LPLs) of the gut in cART-treated HIV-1-positive patients with long-term virologic suppression.

## 2. Results

### 2.1. Demographic and Clinical Characteristics of HIV-1-Positive Patients

All study participants were HIV-1-positive Caucasian men with a median age of 42 years (IQR 22–53 years). They initiated therapy during chronic HIV-1 infection and had been on cART for a median of six years (IQR, 1.75 to 16.25 years). The median of CD4^+^ T cell count pre-therapy was 255 cells/mm^3^ (IQR, 42.75–406.75 cells/mm^3^), HIV-1 RNA copies median value was 5.0 Log/mL (IQR, 4.81–5.61 Log/mL). All subjects had been virologically suppressed (<37 HIV-1 RNA copies/mL) for at least one year, and their median CD4^+^ T cell count was 674 (IQR, 564–824 cells/mm^3^) and 683 (IQR, 610–818 cells/mm^3^) cells/mm^3^, respectively, before and after probiotic supplementation of cART. HIV-1 RNA and a search for neurotropic viruses were negative in all CSF samples.

To better understand the improvement in neurocognitive functions in HIV-1-positive patients after probiotic supplementation, we evaluated changes in IDO mRNA and neopterin levels, before (T0), and after six months (T6) of supplementation with probiotics.

### 2.2. IDO mRNA Expression

**Figure 1:**
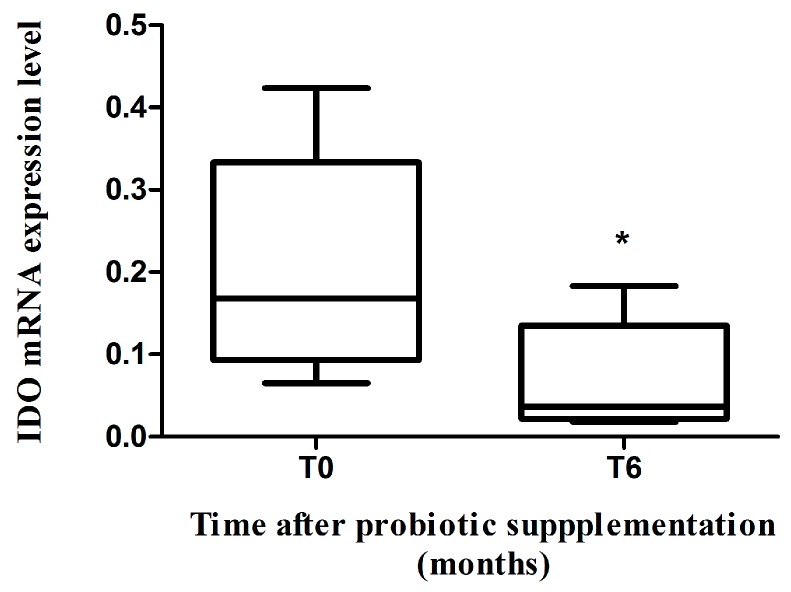
Modulation of indolamine-2,3-dioxygenase (IDO) mRNA in gut-associated lymphoid tissue (GALT). IDO mRNA expression level before (T0) and after six months (T6) of probiotic supplementation in GALT of combined antiretroviral therapy (cART)-treated HIV-1-positive patients with suppressed HIV-1 viremia (*n* = 10). * *p* = 0.04.

We evaluated the expression level of IDO mRNA (which is involved in tryptophan metabolism) in GALT, measuring its amount in LPL. We found that patients with HIV-1 infection had highly variable levels of IDO mRNA before and after probiotic supplementation (coefficient of variability >100%). Following probiotic supplementation, a significantly reduction in the IDO mRNA expression was recorded (*p* = 0.04, Figure 1). 

### 2.3. Neopterin Levels

**Figure 2:**
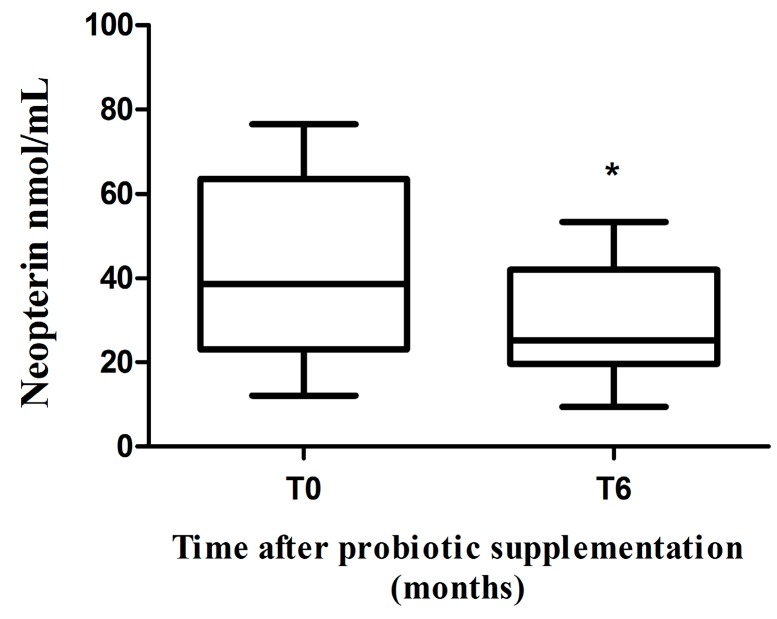
Modulation of neopterin levels in cerebrospinal fluid (CSF). Neopterin expression level before (T0) and after six months (T6) of probiotic supplementation in CSF of cART-treated HIV-1-positive patients with suppressed HIV-1 viremia (*n* = 10). * *p* < 0.004.

In order to evaluate the rate of inflammation in the central nervous system (CNS), we measured neopterin levels in the CSF of HIV-1-positive patients, before (T0) and after (T6) probiotic supplementation. We found higher levels of neopterin at T0 compared to those recorded at T6. In particular, a significant decrease of neopterin levels after 6 months of probiotic supplementation was observed (*p* = 0.004, Figure 2).

Moreover, we investigated the relationship between IDO mRNA expression in the GALT and the level of neopterin in the CSF. We found that at T0 there is a positive correlation between IDO mRNA expression and neopterin level, while at T6, the correlation is no longer observed (Table 1).

## 3. Discussion

In this study, we focused on the relationship between the rate of CSF inflammation measured in terms of neopterin levels and the GALT-associated expression of IDO mRNA in HIV-1-positive patients with suppressed HIV-1 viremia under treatment with cART, before and after supplementation with a high-dose multi-strain probiotic. The high levels of both neopterin and IDO mRNA observed at the enrollment cannot be explained by the HIV-1 RNA levels, because all patients had undetectable HIV-1 RNA levels, both in peripheral blood and in CSF. 

It is known that the introduction of cART reduces the effects of HIV-1 on the neurological impairment, but does not normalize them. To date, the main monocyte activation marker known regarding CNS inflammation in HIV-1 infection is CSF neopterin [22]. In particular, neopterin is a sensitive marker of immune activation, and it is produced by monocyte-derived cells [23]. Neopterin detected in the CSF is originated in the CNS [24], and during HIV-1 infection is expressed at high levels, especially in patients with HAND [25]. Despite cART, neopterin persists at higher levels in HIV-1-positive patients compared to healthy controls, although an early decrease of its amount in CSF is observed after initiation of therapy [23]. These data suggest an ongoing HIV-1-related pathological process within the CNS, where a continuous neuroinflammation and axonal degradation occurs, despite cART [24]. Moreover, enhanced cell trafficking (monocyte-derived cells) from the periphery can enhance CNS infection and neuroinflammation, and are involved in the increased blood–brain barrier (BBB) permeability, while is not clear if neopterin levels are associated with the BBB permeability during HIV-1 infection [22]. However, the amount of neopterin levels is considered a parameter to monitor the activation of immune system [26,27]. Other studies reported a correlation between CSF neopterin levels and CSF neurofilament light protein (NFL)—a biomarker of neuronal injury—in cART-treated HIV-1-positive patients [23]. Consequently, we chose to measure the neopterin levels in CSF as a marker of the expression of brain immune activation, and our findings show a significant decrease in CSF neopterin levels recorded in all HIV-1-positive patients after 6 months of high concentration multistrain probiotic supplementation. From these data, we could speculate the benefit of this high-concentration multistrain probiotic supplementation on the CNS inflammation in treated HIV-1-positive patients and confirmed the role of neopterin as a biomarker of immune activation in CSF. We also hypothesize that the neopterin level measured in the CSF is related to the gut flora of the HIV-1-positive patients. In this regard, a fair number of studies have observed the correlation between gut and brain [28,29]. Cryan et al. describes the gastrointestinal (GI) tract like the scaffold that links the CNS and gut microbiota [28]. Several conditions, such as stress, depression and HIV-1 infection, can modify the bidirectional communication between CNS and gut [30]. In this regard, it is well known that dysbiosis occurs in the earliest phase of the HIV-1 infection, and it is able to affect intestinal permeability [31,32,33,34] and mucosal immunity [35,36,37]. The mechanisms by which the microbiota affects CNS function in HIV-1-positive subjects are various and still poorly understood. They include inflammation and the alterations of immune activation that can directly affect the brain through the production of cytokines, microbial metabolites (e.g., short-chain fatty acids: n-butyrate, acetate, and propionate) and through the alteration of tryptophan metabolism [29]. Tryptophan—an essential amino acid introduced by diet—is absorbed in the gut, enters the circulation, and through the BBB, arrives in the CNS [38,39]. A critical role in this context seems to be played by IDO; this enzyme induced by many cytokines like IFN β and IFN γ, as well as by HIV-1—metabolizes a large part of the tryptophan, generating kinurenine and some other metabolites. Quinlinic acid has been associated with neuronal toxicity, lesions of the central nervous system, and neurocognitive disorders in HIV-1-positive patients [2,10,11,12,13]. The dark side of IDO activity is a deregulation of the Th17/Treg cells ratio, with a shift toward a tolerogenic immunity and a blunted T cell-mediated effector immunity, contributing to the depletion of CD4^+^ T cells [6].

Our results show that probiotic supplementation of cART significantly reduces IDO mRNA expression in the GALT. A positive correlation was also recorded between GALT-associated IDO mRNA and CSF neopterin levels before probiotic supplementation. We speculate that IDO activity and tryptophan metabolism are involved in the inflammation that occurs in the CNS, actively participating in the onset of neurocognitive disorders observed during HIV-1 infection. Notably, a significant reduction of both compounds after six months of probiotic administration has been recorded, which can open the door to the management of neurological complications in people living with HIV-1 infection if confirmed by a larger study. Indeed, HIV-1 may penetrate inside the CNS alone and through monocyte binding, cells which are able to cross the BBB [22]. The brain is an important reservoir of HIV-1, but it remains unclear if the virus causes of persistent low-grade inflammation during cART, or if the virus—after evolution and adaptation to the environment—drives an inflammatory response in the brain [40,41]. The pivotal role of inducing a status of activation in the CNS is attributed to monocyte-derived macrophages; monocytes entering the CNS differentiate into macrophages, and during activation can produce several substances important in the pathogenesis of neurocognitive disorders [42,43]. The opportunity to reduce the activation of the IDO pathway through this probiotic product could break the vicious gut–brain circle and in this manner prevent the neurocognitive impairment observed in HIV-1-positive patients.

Even though we found a correlation between IDO expression and neopterin levels prior to supplementation with probiotics, after 6 months of probiotics supplementation, this correlation was lost. We attributed this unexpected result to the lack of statistical power due to the small sample size of patients analyzed.

This study was limited by the small sample size of patients analyzed, by the lack of evaluation of IDO enzyme activity and tryptophan metabolites in the blood, and by a full assessment of liver function. In fact, we evaluated only IDO mRNA expression in the GALT—which reflects the local rate of activity of this key enzyme involved in tryptophan metabolism—but we were unable to assess the “origin” of the high levels quinolinic acid at the enrollment. Furthermore, since tryptophan metabolism has been indicated as the product of pathogenic gut bacteria [16], and since a decreased IDO1 activity associated with a preservation of Th17 has recently been observed in chronic simian immunodeficiency virus (SIV) infection after probiotic supplementation [44], it could be also important to look for changes in the gut microbiome.

Finally, in consideration of the unregulated probiotic market and of the seriousness of the HIV infection, it should be kept in mind that our results were obtained with a specific product and may not apply to different probiotic products

## 4. Materials and Methods

### 4.1. Patients

Ten HIV-1-positive patients successfully treated with cART were recruited at the Division of Infectious Diseases, Department of Public Health and Infectious Diseases, Hospital of “Sapienza” University of Rome (Italy).

The study was approved by the institutional review board (Department of Public Health and Infectious Diseases, “Sapienza” University of Rome and the Ethics Committee of Umberto I General Hospital, Rome, 2013). All study participants signed written informed consent. The inclusion criteria used to enroll HIV-positive patients were the following: (i) individuals signed the informed consent; (ii) women or men at least 18 years of age; (iii) in highly-active ART (HAART) with HIV RNA <37 copies/mL and CD^4^ T cell counts >400 cells/mm^3^. Exclusion criteria were the following: (i) patients with known allergy or intolerance to probiotics; (ii) diarrhea; (iii) history of or current inflammatory diseases of the small intestine or drug addiction; (iv) use of antibiotics or probiotics during the 3 weeks prior the enrollment; (v) pregnancy. All HIV-1-positive patients received a high-concentration lyophilized multistrain probiotic powder supplement twice a day for six months. The probiotic preparation daily dosage was 1.8 × 10^12^ live bacteria, according to the previously reported studies [45,46]. The product containing the same strains present in the preparation employed in our study (*Lactobacillus plantarum* DSM 24730, *Streptococcus thermophilus* DSM 24731, *Bifidobacterium breve* DSM 24732, *Lactobacillus paracasei* DSM 24733, *Lactobacillus delbrueckii* subsp. *bulgaricus* DSM 24734, *Lactobacillus acidophilus* DSM 24735, *Bifidobacterium longum* DSM 24736, *Bifidobacterium infantis* DSM 24737) currently sold under the brand Vivomixx^®^ in Continental Europe and Visbiome^®^ in USA and Canada. 

### 4.2. Samples

Patients were sampled for peripheral blood and colonoscopy before and after probiotic supplementation. Colonic washing was carried out by polyethylene glycol-electrolyte solution (PEG) administration 24 h before the examination. The endoscopic procedure was performed with conscious sedation (midazolam 5 mg/iv) using large cup forceps (Radial Jaw 4, Boston Scientific, Natick, MA, USA). All HIV-1-positive patients underwent a total colonoscopy and retrograde ileoscopy for at least 10 cm of distal ileum with conventional or slim scope (model CF or PCF-160 AI, Olympus Medical Europe GmbH, Hamburg, Germany). We obtained specimens (two biopsies from each site) from the terminal ileum, cecum, ascending, transverse, and descending colon.

Lamina propria lymphocytes (LPLs) was stored as dried pellets for RNA extraction. Gut biopsies from each intestine site were pooled and processed. Briefly, biopsies collected in RPMI 1640 were washed twice with EDTA wash media, resuspended, and incubated for 1 h at room temperature in EDTA solution 5 mM. Supernatant containing intraepithelial lymphocytes was removed, and biopsies were digested by 1 h incubation at 37 °C with 1 mg/mL collagenase (Sigma-Aldrich, Milan, Italy) and 1.5 U DNAse I (Sigma-Aldrich), allowing the isolation of LPLs that were filtered through a 70 µm cell strainer. 

CSF was collected by lumbar puncture, centrifuged, and cell-free supernatant samples were stored in aliquots at −80 °C.

### 4.3. ELISA Assay

CSF Neopterin levels of 10 HIV-1-positive patients were determined by a commercially available solid phase enzyme-linked immunosorbent assay (ELISA) based on the basic principle of a competitive ELISA (IBL International GmbH, Hamburg, Germany).

### 4.4. Real-Time PCR

Quantitative real-time PCR for IDO mRNA expression was carried out with the LightCycler 480 instrument (Roche, Basel, Switzerland). Briefly, total RNA was extracted from LPLs using the RNeasy Plus Universal Tissue Mini Kit (Invitrogen, Carlsbad, CA, USA) and reverse transcribed using the High-Capacity cDNA Reverse Transcription Kit (Applied Biosystem, Foster City, CA, USA), according to the manufacturer’s protocol. Primers and probes for each gene were added to the Probes Master Mix (Roche, Basel, Switzerland) at 500 and 250 nM, respectively, in a final volume of 20 µL. The housekeeping gene β-glucuronidase was used as an internal control. Gene expression values were calculated by the comparative Ct method. The primers and probe sequences used for IDO were the following (Forward 5′-GCATTTTTCAGTGTTCTTCGCA-3′, Reverse 5′-CATACACCAGACCGTCTGATAGCT-3′, Probe5′-(6FAM) ATATTTGTCTGGCTGGAAAGGCAACCCC (TAM)-3′) [47].

### 4.5. Statistical Analysis

Statistical analyses and graphic presentation were done on CSF and gut samples of 10 HIV-1-positive patients. In particular, neopterin determination and linear regression with Spearman’s correlation coefficient were calculated using GraphPad Prism software, version 5.0 (GraphPad Software Inc., La Jolla, CA, USA), and data were compared by Wilcoxon test for paired samples. IDO mRNA expression was analyzed using Primer-E software, version 6.0 (PRIMER-E Ltd., Lutton, UK), and data were compared using *t*-test. Differences were considered statistically significant when *p* < 0.05.

## 5. Conclusions

In conclusion, the probiotic product employed in our study, when supplemented for 6 months in HIV-1-positive patients, is associated with a significant reduction of the CSF neopterin and GALT-associated IDO mRNA levels. Since the results for the use of adjunctive therapies to reduce the levels of immune activation markers in CSF have been disappointing so far, our pilot study showing the efficacy of this specific probiotic product should be followed by a larger confirmatory trial.

## Figures and Tables

**Table 1:** Correlation between cerebrospinal fluid (CSF) neopterin levels and gut-associated lymphoid tissue (GALT)-associated IDO mRNA expression before (T0) and after six months (T6) of probiotic supplementation in cART-treated HIV-1-positive patients with suppressed HIV-1 viremia (*n* = 10).

	IDO mRNA T0	IDO mRNA T6
Neopterin T0	*p* = 0.004; *r* = 0.94	NA
Neopterin T6	NA	*p* = 0.54; *r* = −0.31

Spearman’s rho coefficient was used to assess the correlations between neopterin and IDO levels; significant correlations are highlighted in bold. NA = not applicable.

## References

[1] Routy J.P., Mehraj V., Vyboh K., Cao W., Kema I., Jenabian M.A. (2015). Clinical relevance of kynurenine pathway in HIV/AIDS: An immune checkpoint at the crossroads of metabolism and inflammation. AIDS Rev..

[2] Vyboh K., Jenabian M.A., Mehraj V., Routy J.P. (2015). HIV and the gut microbiota, partners in crime: Breaking the vicious cycle to unearth new therapeutic targets. J. Immunol. Res..

[3] Carabotti M., Scirocco A., Maselli M.A., Severi C. (2015). The gut-brain axis: Interactions between enteric microbiota, central and enteric nervous systems. Ann. Gastroenterol..

[4] Montiel-Castro A.J., González-Cervantes R.M., Bravo-Ruiseco G., Pacheco-López G. (2013). The microbiota-gu-brain axis: Neurobehavioral correlates, health and sociality. Front. Integr. Neurosci..

[5] Atarashi K., Tanoue T., Shima T., Imaoka A., Kuwahara T., Momose Y., Cheng G., Yamasaki S., Saito T., Ohba Y. (2011). Induction of colonic regulatory T cells by indigenous Clostridium species. Science.

[6] Favre D., Mold J., Hunt P.W., Kanwar B., Loke P., Seu L., Barbour J.D., Lowe M.M., Jayawardene A., Aweeka F. (2010). Tryptophan catabolism by indoleamine 2,3-dioxygenase 1 alters the balance of TH17 to regulatory T cells in HIV disease. Sci. Transl. Med..

[7] Serrano-Villar S., Rojo D., Martínez-Martínez M., Deusch S., Vázquez-Castellanos J.F., Sainz T., Vera M., Moreno S., Estrada V., Gosalbes M.J. (2016). HIV infection results in metabolic alterations in the gut microbiota different from those induced by other diseases. Sci. Rep..

[8] Boasso A. (2011). Wounding the immune system with its own blade: HIV-induced tryptophan catabolism and pathogenesis. Curr. Med. Chem..

[9] Boasso A., Shearer G.M., Chougnet C. (2009). Immune dysregulation in human immunodeficiency virus infection: Know it, fix it, prevent it?. J. Intern. Med..

[10] Heyes M.P., Brew B.J., Martin A., Price R.W., Salazar A.M., Sidtis J.J., Yergey J.A., Mouradian M.M., Sadler A.E., Keilp J. (1991). Quinolinic acid in cerebrospinal fluid and serum in HIV-1 infection: Relationship to clinical and neurological status. Ann. Neurol..

[11] Heyes M.P., Ellis R.J., Ryan L., Childers M.E., Grant I., Wolfson T., Archibald S., Jernigan T.L., HNRC Group, HIV Neurobehavioral Research Center (2001). Elevated cerebrospinal fluid quinolinic acid levels are associated with region-specific cerebral volume loss in HIV infection. Brain.

[12] Valle M., Price R.W., Nilsson A., Heyes M., Verotta D. (2004). CSF quinolinic acid levels are determined by local HIV infection: Cross-sectional analysis and modelling of dynamics following antiretroviral therapy. Brain.

[13] O’Mahony S.M., Clarke G., Borre Y.E., Dinan T.G., Cryan J.F. (2015). Serotonin, tryptophan metabolism and the brain-gut-microbiome axis. Behav. Brain Res..

[14] Boasso A., Herbeuval J.P., Hardy A.W., Anderson S.A., Dolan M.J., Fuchs D., Shearer G.M. (2007). HIV inhibits CD4^+^ T-cell proliferation by inducing indoleamine 2,3-dioxygenase in plasmacytoid dendritic cells. Blood.

[15] Reeves R.K., Rajakumar P.A., Evans T.I., Connole M., Gillis J., Wong F.E., Kuzmichev Y.V., Carville A., Johnson R.P. (2011). Gut inflammation and indoleamine deoxygenase inhibit IL-17 production and promote cytotoxic potential in NKp44^+^ mucosal NK cells during SIV infection. Blood.

[16] Vujkovic-Cvijin I., Dunham R.M., Iwai S., Maher M.C., Albright R.G., Broadhurst M.J., Hernandez R.D., Lederman M.M., Huang Y., Somsouk M. (2013). Dysbiosis of the gut microbiota is associated with HIV disease progression and tryptophan catabolism. Sci. Transl. Med..

[17] Griffin D.E., McArthur J.C., Cornblath D.R. (1991). Neopterin and interferon-gamma in serum and cerebrospinal fluid of patients with HIV-associated neurologic disease. Neurology.

[18] Sönnerborg A.B., von Stedingk L.V., Hansson L.O., Strannegård O.O. (1989). Elevated neopterin and β2-microglobulin levels in blood and cerebrospinal fluid occur early in HIV-1 infection. AIDS.

[19] Fuchs D., Chiodi F., Albert J., Asjö B., Hagberg L., Hausen A., Norkrans G., Reibnegger G., Werner E.R., Wachter H. (1989). Neopterin concentrations in cerebrospinal fluid and serum of individuals infected with HIV-1. AIDS.

[20] Eden A., Price R.W., Spudich S., Fuchs D., Hagberg L., Gisslén M. (2007). Immune activation of the central nervous system is still present after >4 years of effective HAART. J. Infect. Dis..

[21] Yilmaz A., Yiannoutsos C.T., Fuchs D., Price R.W., Crozier K., Hagberg L., Spudich S., Gisslén M. (2013). Cerebrospinal fluid neopterin decay characteristics after initiation of antiretroviral therapy. J. Neuroinflamm..

[22] Hagberg L., Cinque P., Gisslen M., Brew B.J., Spudich S., Bestetti A. (2010). Cerebrospinal fluid neopterin: An informative biomarker of central nervous system immune activation in HIV-1 infection. AIDS Res. Ther..

[23] Calcagno A., Atzori C., Romito A., Vai D., Audagnotto S., Stella M.L., Montrucchio C., Imperiale D., di Perri G., Bonora S. (2016). Blood brain barrier impairment is associated with cerebrospinal fluid markers of neuronal damage in HIV-positive patients. J. Neurovirol..

[24] Edén A., Marcotte T.D., Heaton R.K., Nilsson S., Zetterberg H., Fuchs D., Franklin D., Price R.W., Grant I., Letendre S.L. (2016). Increased intrathecal immune activation in virally suppressed HIV-1 infected patients with neurocognitive impairment. PLoS ONE.

[25] Andersson L.M., Hagbwerg L., Fuchs D., Svennerholm B., Gisslen M. (2001). Increased blood brain-barrier permeability in neuroasymptomatic HIV-1-infected individuals-correlation with cerebrospinal fluid HIV-1 RNA and neopterin levels. J. Neurovirol..

[26] Schroecksnadel K., Sarcletti M., Winkler C., Mumelter B., Weiss G., Fuchs D., Kemmler G., Zangerle R. (2008). Quality of life and immune activation in patients with HIV-infection. Brain Behav. Immun..

[27] Huber C., Batchelor J.R., Fuchs D., Hausen A., Lang A., Niederwieser D., Reibnegger G., Swetly P., Troppmair J., Wachter H. (1984). Immune response-associated production of neopterin. Release from macrophages primarily under control of interferon-γ. J. Exp. Med..

[28] Borre Y.E., Moloney R.D., Clarke G., Dinan T.G., Cryan J.F. (2014). The impact of microbiota on brain and behavior: Mechanisms & therapeutic potential. Adv. Exp. Med. Biol..

[29] Cryan J.F., Dinan T.G. (2012). Mind-altering microorganisms: The impact of the gut microbiota on brain and behaviour. Nat. Rev. Neurosci..

[30] Mayer E.A. (2011). Gut feelings: The emerging biology of gut–brain communication. Nat. Rev. Neurosci..

[31] Frazier T.H., DiBaise J.K., McClain C.J. (2011). Gut microbiota, intestinal permeability, obesity-induced inflammation, and liver injury. J. Parenter. Enter. Nutr..

[32] Camilleri M., Lasch K., Zhou W. (2012). Irritable bowel syndrome: Methods, mechanisms, and pathophysiology. The confluence of increased permeability, inflammation, and pain in irritable bowel syndrome. Am. J. Physiol. Gastrointest. Liver Physiol..

[33] Matricon J., Meleine M., Gelot A., Piche T., Dapoigny M., Muller E., Ardid D. (2012). Review article: Associations between immune activation, intestinal permeability and the irritable bowel syndrome. Aliment. Pharmacol. Ther..

[34] Simrén M., Barbara G., Flint H.J., Spiegel B.M., Spiller R.C., Vanner S., Verdu E.F., Whorwell P.J., Zoetendal E.G., Rome Foundation Committee (2013). Intestinal microbiota in functional bowel disorders: A Rome foundation report. Gut.

[35] Round J.L., Mazmanian S.K. (2009). The gut microbiota shapes intestinal immune responses during health and disease. Nat. Rev. Immunol..

[36] Ringel Y., Maharshak N. (2013). Intestinal microbiota and immune function in the pathogenesis of irritable bowel syndrome. Am. J. Physiol. Gastrointest. Liver Physiol..

[37] Hughes P.A., Zola H., Penttila I.A., Blackshaw L.A., Andrews J.M., Krumbiegel D. (2013). Immune activation in irritable bowel syndrome: Can neuroimmune interactions explain symptoms?. Am. J. Gastroenterol..

[38] Palego L., Betti L., Rossi A., Giannaccini G. (2016). Tryptophan biochemistry: Structural, nutritional, metabolic, and medical aspects in humans. J. Amino Acids.

[39] Murray M.F. (2010). Insights into therapy: Tryptophan oxidation and HIV infection. Sci. Transl. Med..

[40] Alexaki A., Liu Y., Wigdahl B. (2008). Cellular reservoirs of HIV-1 and their role in viral persistence. Curr. HIV Res..

[41] Coleman C.M., Wu L. (2009). HIV interactions with monocytes and dendritic cells: Viral latency and reservoirs. Retrovirology.

[42] Epelman S., Lavine K.J., Randolph G.J. (2014). Origin and functions of tissue macrophages. Immunity.

[43] Distrutti E., O’Reilly J.A., McDonald C., Cipriani S., Renga B., Lynch M.A., Fiorucci S. (2014). Modulation of intestinal microbiota by the probiotic VSL#3 resets brain gene expression and ameliorates the age-related deficit in LTP. PLoS ONE.

[44] Vujkovic-Cvijin I., Swainson L.A., Chu S.N., Ortiz A.M., Santee C.A., Petriello A., Dunham R.M., Fadrosh D.W., Lin D.L., Faruqi A.A. (2015). Gut-resident lactobacillus abundance associates with IDO1 inhibition and Th17 dynamics in SIV-infected macaques. Cell Rep..

[45] Mardini H.E., Grigorian A.Y. (2014). Probiotic mix VSL#3 is effective adjunctive therapy for mild to moderately active ulcerative colitis: A meta-analysis. Inflamm. Bowel. Dis..

[46] Kumar R., Singh J. (2013). The emerging therapy with probiotics in the management of inflammatory bowel disease: Current status. Int. J. Basic Clin. Pharmacol..

[47] Scagnolari C., Monteleone K., Selvaggi C., Pierangeli A., D’Ettorre G., Mezzaroma I., Turriziani O., Gentile M., Vullo V., Antonelli G. (2016). ISG15 expression correlates with HIV-1 viral load and with factors regulating T cell response. Immunobiology.

